# Human Herpesviruses Increase the Severity of Hepatitis

**DOI:** 10.3390/biology10060483

**Published:** 2021-05-29

**Authors:** Kirill I. Yurlov, Olga V. Masalova, Lidiia B. Kisteneva, Irina N. Khlopova, Evgeny I. Samokhvalov, Valentina V. Malinovskaya, Vladimir V. Parfyonov, Alexander N. Shuvalov, Alla A. Kushch

**Affiliations:** 1 Gamaleya National Research Center for Epidemiology and Microbiology, Ministry of Health of the Russian Federation, Moscow 123098, Russia; kir34292@yandex.ru (K.I.Y.); lborisovna@yandex.ru (L.B.K.); khlopova.ira@yandex.ru (I.N.K.); e_samokh@hotmail.com (E.I.S.); vvm@viferon.su (V.V.M.); parfvlad@yandex.ru (V.V.P.); shuvalovan@viferon.su (A.N.S.); vitallku@mail.ru (A.A.K.); 2 Department of Health, Moscow Infectious Clinical Hospital No. 1, Moscow 125367, Russia

**Keywords:** viral hepatitis, hepatitis C virus, unspecified hepatitis, herpesviruses, viral DNA, HCV RNA, disease severity, liver cirrhosis

## Abstract

**Simple Summary:**

More than 300 million people worldwide suffer from hepatitis B or hepatitis C and more than 1 million people die each year from cirrhosis and liver cancer. In some cases, the nature of hepatitis remains unclear. The purpose of this research was to assess the prevalence of human herpesviruses (cytomegalovirus, Epstein–Barr virus, and herpesvirus type 6) in patients with hepatitis, and to examine their effect on the disease severity. In the clinical materials of 377 patients with acute or chronic hepatitis, DNA of these three herpesviruses was detected in the blood in 13.5% of patients with viral hepatitis B or C and in 10.1% of patients with hepatitis of unspecified etiology. The cirrhosis was diagnosed in patients with herpesviruses 3 times more often than in patients without them. In patients with hepatitis C, the incidence of herpesviruses was higher in the tissue samples of liver biopsies (38.7%) than in the blood. Clinical and virological indicators of hepatitis were considerably higher in the patients with coinfection. Since in patients with hepatitis the presence of herpesviruses is associated with a more severe course of the disease, the detection, and herpesvirus DNA monitoring will help to adjust the course of therapy.

**Abstract:**

Acute and chronic liver diseases are a major global public health problem; nevertheless, the etiology of 12–30% of cases remains obscure. The purpose of this research was to study the incidence of human herpesviruses (HHVs) cytomegalovirus, Epstein–Barr virus (EBV), and HHV-6 in patients with hepatitis and to examine the effect of HHV on the disease severity. We studied the clinical materials of 259 patients with hepatitis treated in Infectious Clinic n.1 (Moscow) and the archived materials of 118 patients with hepatitis C. HHV DNA was detected in the whole blood in 13.5% of patients with hepatitis B or C and in 10.1% of patients with hepatitis of unspecified etiology. EBV demonstrated the highest incidence (58.1%). Cirrhosis was diagnosed in 50% of patients with HHV and in 15.6% of patients without HHV. In patients with hepatitis C, the frequency of HHV was higher in liver biopsy (38.7%) compared to blood. The clinical and virological indicators of hepatitis were considerably higher in patients with coinfection. Conclusion: HHV detected in patients with viral hepatitis has been associated with a significant effect on the severity of the disease, and we suggest monitoring HHV DNA in patients with severe hepatitis and/or poor response to antiviral drugs.

## 1. Introduction

Acute and chronic liver diseases are a major global public health problem. As a rule, the causative agents of liver diseases are hepatotropic viruses of hepatitis A, B, C, D, and E. Hepatitis A and E are transmitted by the fecal–oral route and typically end in recovery. The prognosis is favorable, and liver function is normally fully restored. Hepatitis B and C are transmitted parenterally, and acute hepatitis B and C may become chronic with a high risk of a lethal outcome due to liver cirrhosis and hepatocarcinoma. Hepatitis D virus occurs as a coinfection with the hepatitis B virus (chronic hepatitis B with delta agent and delta superinfection of hepatitis B) and is considered to be the most severe form of chronic viral hepatitis [1].

There is a sufficiently effective vaccine against hepatitis B; however, there is no vaccine against hepatitis C. The use of direct-acting antivirals (DAAs) in recent decades allowed for successful treatment of more than 95% of patients with hepatitis C [2]. However, the disappearance of hepatitis C virus (HCV) RNA from the peripheral blood (PB) does not completely exclude an occult infection in the liver [3]. Moreover, despite a high rate of cure, treatment failures can occur in about 3–5% of treated patients due to a high frequency of resistance-associated substitutions. These patients should be identified and receive a triple DAA combination regimen [2].

Inflammatory processes in the liver, in addition to hepatotropic viruses, may cause autoimmune disorders, alcohol intoxication, and fatty liver formation as may certain medications and other, non-hepatotropic, viruses. Among the latter, special attention should be paid to herpesviruses, some of which have been found in hepatitis [4,5,6].

Human diseases are caused by nine herpesviruses, belonging to the Herpesviridae family. Human herpesviruses (HHV) are widespread in the human population, affect various organs and tissues, may cause acute forms of the disease during primary infection, and may transition into a latent state under the influence of effective immunity. The transition of herpesvirus infection from the latent form to reactivation poses a danger, since many causes and mechanisms of this phenomenon are currently poorly understood. The studies aimed at identification of the role of HHV in inflammatory liver diseases are few and contradictory.

This applies not only to hepatitis where the viral etiology is established but also to hepatitis where the origin cannot be determined—so-called “hepatitis of unclear or unspecified etiology”. According to the available data, in acute hepatic failure, hepatitis of unknown origin accounts for 12% [7]. Other authors consider that the cause of fulminant hepatitis cannot be determined in 15–30% of cases [8]. The question of whether HHV may cause acute or chronic liver disease remains controversial.

The aim of this work was to study the spread of 3 HHV (cytomegalovirus (CMV), Epstein–Barr virus (EBV), and human herpesvirus of type 6 (HHV-6)) in patients with viral hepatitis and in patients with hepatitis of unspecified etiology and to assess the HHV impact on the severity of the disease and its outcomes. 

## 2. Materials and Methods

### 2.1. Patients

The study included 259 patients with preliminary diagnoses of acute or chronic hepatitis, who were being examined and treated in the Infectious Clinical Hospital No. 1 of the Moscow Department of Health (ICH 1 of MDH) in 2018–2020. Peripheral blood samples were obtained from all patients.

A retrospective study of clinical materials obtained from 97 patients with chronic hepatitis C (CHC) and from 21 patients with acute hepatitis C (AHC), observed in ICH 1 of MDH in the period 2003–2005, was conducted.

The age of patients ranged from 18 to 71 years with a mean age of 45.1 ± 15.1 years, and men accounted for 59%.

All patients with viral hepatitis were treated with basic and antiviral drugs. Basic therapy included detoxification and the use of hepatoprotective agents. Antiviral therapy included the use of PEG-IFN + ribavirin in 2003–2005 or DAA in 2018–2020 in the case of viral hepatitis C, and nucleoside inhibitors in the case of viral hepatitis B in accordance with [9,10,11,12,13,14]. Prior to the study, informed written consent was obtained from each patient. The study procedures were performed according to the Declaration of Helsinki and approved by the local Ethics Committee of the Infectious Clinical Hospital No. 1 of the Moscow Department of Health (No. 16, 25. 12. 2017).

### 2.2. Clinical Materials

Peripheral blood (PB) samples were immediately delivered to the research laboratory. Peripheral blood mononuclear cells (PBMCs) isolated in the Ficoll gradient (Ficoll-Paque, “Pharmacia”, Sweden), and liver needle biopsy samples were stored at −20 °C without repeated freeze–thaw cycles.

All patients were examined using standard clinical, biochemical, and instrumental methods.

### 2.3. Differential Diagnostics for Viral Hepatitis

The diagnosis was established in accordance with the International Classification of Diseases of the 10th revision (ICD-10) on the basis of generally accepted epidemiological, virological, and clinical-laboratory criteria, and on the basis of the exclusion of other etiological factors in accordance with national standards and World Health Organization (WHO) recommendations [15]. Serological determination of markers of viral hepatitis A, B, C, D, and E (anti-HAV-IgM, HBsAg, HBeAg, anti-HCV IgG, anti-HBcore IgM and IgG, anti-HDV IgM and IgG, and anti-HEV-IgM) was performed using ELISA and chemiluminescence methods (Roche Cobas e8000 602, “Roche Diagnostics”, Switzerland). Hepatitis B virus (HBV) DNA, HCV RNA, and HDV RNA were analyzed using PCR and real-time RT-PCR (“InterLabService”, Moscow, Russian Federation). Patients with the diagnoses of autoimmune, alcoholic, toxic hepatitis, and steatohepatitis were excluded.

### 2.4. Human Herpesvirus (HHV) Detection

HHV DNA was detected by real-time PCR using a PCR reagent kit, “AmpliSensEBV/CMV/HHV6-screen-FL”, “InterLabService”, Moscow, Russian Federation. The kit allows the simultaneous detection of the DNA of three HHVs: EBV, CMV, and HHV-6. The β-globin gene was used as an endogenous internal control. The limit of detection was 100 copies/mL.

### 2.5. Study Design for Chronic Hepatitis C Patients

The clinical materials from the patients with CHC were studied in greater detail. The spectrum of antibodies to individual HCV proteins and its activity (titers) were determined using the test system “ELISA-anti-HCV-spectrum” (“Diagnostic Systems”, N. Novgorod, Russian Federation).

HCV RNA was determined using the “nested” version of PCR (RT-PCR) using synthetic primers from the 5’-nontranslated region of HCV [16]. HCV replicative (minus strand) RNA was detected in cell lysates using the primers described earlier [17].

The HCV genotype was determined by the method Ohno O. et al. [18].

The concentration of the nucleocapsid (core) protein in blood serum was determined using the ELISA sandwich version, as described earlier [19].

HCV proteins in hepatocytes were detected by indirect immunoperoxidase staining using a mixture of monoclonal antibodies (mAbs) raised against the core (1F9), NS3 (2H4), NS4 (a mixture of 3F12 to NS4A and 6B11 to NS4B proteins), and NS5A (3F4) proteins, as described previously [20]. The cryostat liver sections were stained as described earlier [21] with minor modifications: 3,3’-diaminobenzidine tetrahydrochloride (DAB, “Sigma”, USA) was used as an HPRO substrate instead of 3-amino-9-ethyl-carbazole (AEC). Both the number of stained cells and the intensity of staining were taken into consideration for interpretation of the results.

The liver functional activity was assessed by the level of aminotransferases in the blood serum: alanine aminotransferase (ALT) and aspartate aminotransferase (AST). Liver morphological changes were assessed and scored according to the METAVIR System for the evaluation of histology activity and fibrosis [22].

### 2.6. Statistical Analysis

Statistical analysis was performed with Statistica 8 software (StatSoft Inc., Tulsa, OK, USA). Prism 7 software (GraphPad7, SanDiego, CA, USA) was used to create graphs. Categorical variables, such as sex and the detection rate of different characteristics, were tested using χ2 and Fisher’s exact tests. Continuous variables, such as age, illness duration, serum ALT and AST levels, and the concentration of the HCV core in serum, are presented as the means ±standard errors of means (SEM) and analyzed using a two-tailed Student’s *t*-test. A *p*-value < 0.05 was considered statistically significant.

## 3. Results

### 3.1. The Prevalence of Herpesviruses in Patients with Hepatitis

At the first stage, we studied the frequency of the DNA occurrence of three herpesviruses—EBV, CMV, and HHV-6—in the PB of 259 patients who were under inpatient observation in ICH 1 of MDH in 2018–2020 with viral hepatitis A, B, C, and D, and with a preliminary diagnosis of “hepatitis of unspecified etiology”. Herpesviruses were detected in 12% of patients; the detection rate varied depending on the pathogen etiology; however, the differences did not reach statistical significance (*p* > 0.05, Table 1).

Most often, the EBV genome was detected—58.1% of all the PB samples contained the studied herpesviruses (18/31). The analysis of associations of the etiological factor of hepatitis with the markers of different herpesviruses showed that acute and chronic hepatitis C were associated to a greater extent with EBV, hepatitis B was associated with EBV and HHV-6, while hepatitis of unspecified etiology was associated with EBV and CMV. In the case of hepatitis C, all three studied HHV and herpesviral coinfections were detected in PB. In the case of hepatitis of unspecified etiology, HHV was detected in the PB in 7 out of 69 patients (10.1%), which did not significantly differ in the occurrence frequency from that in cases of viral hepatitis B and C (on average 13.5%).

### 3.2. Clinical Conditions in Patients with Viral Hepatitis in Combination with Herpesvirus Infection were Worse Than Those in Those without It

**Figure 1:**
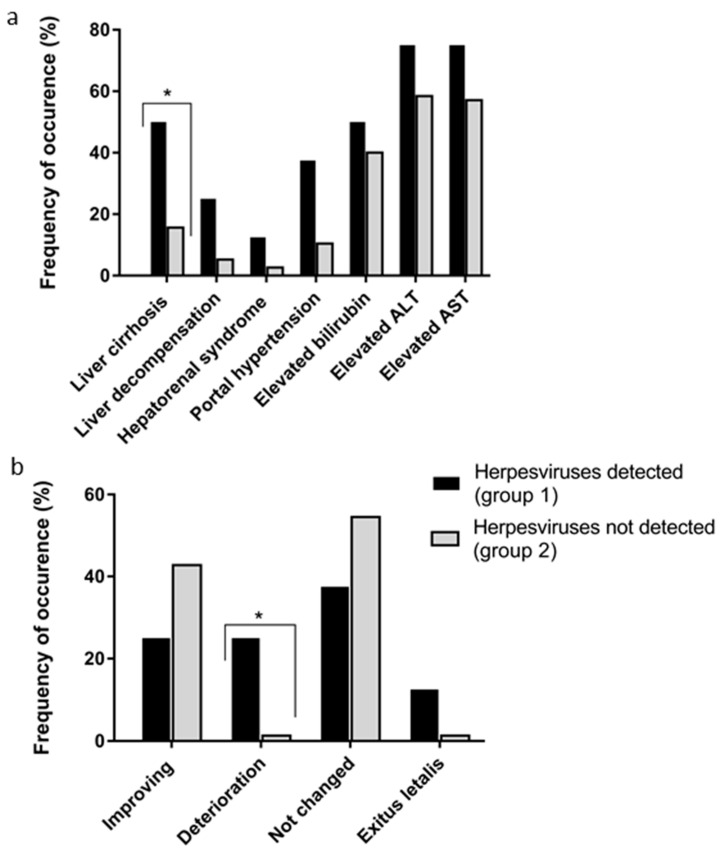
Clinical and laboratory signs (**a**) and clinical condition (**b**) in patients with viral hepatitis and hepatitis of unspecified etiology in coinfection with herpesviruses (group 1, *n* = 8) and without it (*n* = 77, group 2). Values are the detection rates, %. * *p* < 0.05 between groups according χ2 and Fisher’s exact tests. Alanine aminotransferase (ALT) and aspartate aminotransferase (AST).

A more detailed analysis of the clinical and laboratory data from 85 of these patients admitted in 2020, which represented a random sample (32.8%) of all 259 examined patients, was conducted. Markers of persistent herpesvirus infection were found in 8 out of 85 patients with liver injury of different nature. Analysis of the clinical progression of hepatitis in patients with concomitant herpesvirus infection (group 1) and without it (group 2) revealed the more severe development of hepatitis in group 1 (Figure 1a).

Liver cirrhosis is the final pathological result of various chronic liver diseases. It was diagnosed in every second patient with hepatitis in combination with herpesvirus infection and less often in patients without herpesvirus infection (15.6%, *p* < 0.05). Serious syndromes complicating the hepatitis progression, such as liver decompensation, hepatorenal syndrome, and portal hypertension were also more often diagnosed in the patients of group 1 over group 2; however, the differences did not reach statistical significance (see Figure 1a). Increases in the bilirubin, ALT, and AST levels (greater than two times the upper limit of normal) were more often observed in group 1 patients but also did not reach statistical significance. An increase in the number of patients in the compared groups may allow further determination of the significance of the changes introduced by herpesvirus infection into the state of coinfected patients.

All patients with viral hepatitis were treated with basic and antiviral drugs. Improving conditions, i.e., the normalization of the liver enzymes, bilirubin, relief of asthenic syndrome, and normalization of the liver and spleen size were noted in 25% of the patients in group 1 and 42.8% in group 2 (Figure 1b).

Deterioration in the condition (increased symptoms of liver cell necrosis and hepatic failure, and increased levels of liver enzymes) were observed in 25% of the patients with concomitant herpesvirus infection and in only 1.3% without herpesvirus infection (*p* < 0.05). The severe progression of chronic viral hepatitis C in one patient whose PB was found to contain HHV-6 + EBV DNA (from group 1) led to exitus letalis. A fatal outcome was also observed in 1 of 77 patients with liver cirrhosis from group 2.

### 3.3. Comparative Analysis of the Detection Rates of Herpesviruses in Various Biological Materials

More than half of the patients (134 out of 259, 51.7%, Table 1) were infected with HCV and with diagnoses of AHC or CHC. In this regard, we examined this group of patients in more detail. Freshly obtained whole PB and serum or plasma samples from 134 patients were studied, and archival materials from 118 patients: PBMC and liver biopsy samples, stored in frozen form. The comparative frequency of herpesvirus DNA detection in different biological materials in patients with hepatitis C is provided in Table 2. Herpesviruses were the most rarely detected in blood serum (10.9%) and the most often in liver samples (38.7%).

The detection rate for herpesviruses in the liver biopsies was statistically significantly higher than that in other materials (*p* < 0.005), while the differences in detection in the whole blood, PBMCs, and serum were not statistically significant. Herpesvirus DNA was detected more than two-times as often in the liver biopsies of CHC patients, compared with in blood cells where the most significant predominance was observed in relation to HHV-6 (*p* = 0.0001, Table 2). In contrast, EBV was most often detected in the PBMC from the archival materials of patients with CHC or AHC (*p* = 0.03). In other biological materials, herpesviruses were detected with a similar frequency (*p* > 0.05).

### 3.4. Negative Impact of Herpesviruses on the Clinical and Virological Data of CHC Patients

We investigated whether herpesviruses in the blood and liver affected CHC progression. We divided the patients with CHC (*n* = 97), from whom archival materials (liver biopsy or PBMC) were obtained, into two groups, depending on the detection of herpesviruses, and compared the available demographic, clinical, and virological data of the patients.

From the data presented (Table 3), the patients did not differ in age, duration of the disease, and addiction to injectable drugs. Statistically significant differences were found by sex: there were more men among the patients with herpesviruses (group 1) compared with in group 2, in which no herpesviruses were found (*p* < 0.05).

**Figure 2:**
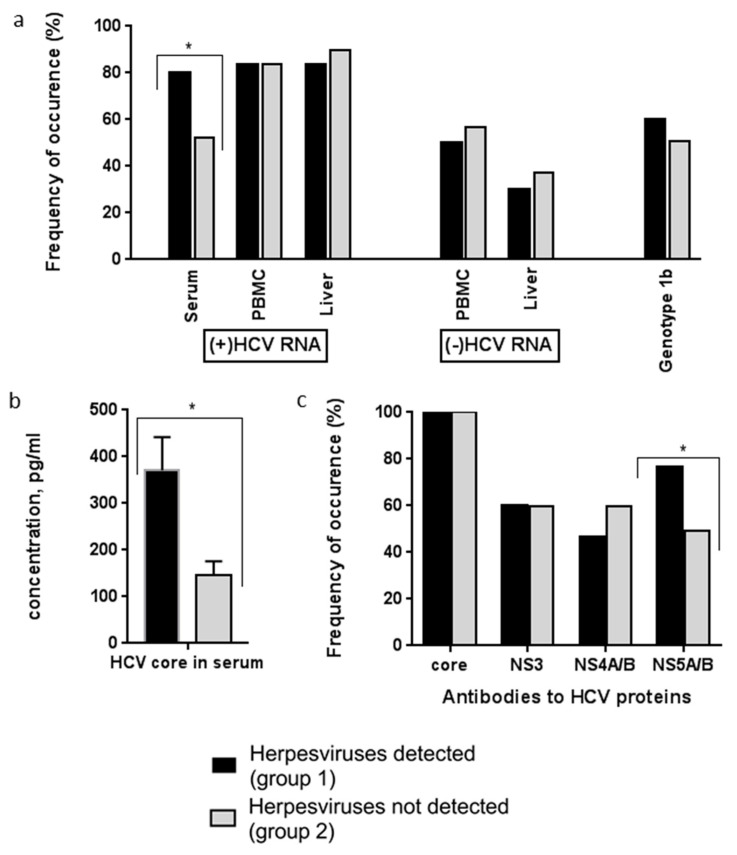
Virological data of patients with chronic hepatitis C depending on the detection of herpesvirus DNA. (**a**) HCV genotype, HCV RNA plus-strand (genomic) and minus-strand (replicative) were detected in peripheral blood and liver by RT-PCR. (**b**) Concentrations of HCV core in sera were estimated by ELISA using original monoclonal antibodies (mAbs). (**c**) The presence of anti-HCV antibodies to viral proteins was determined by indirect ELISA. Group 1 (*n* = 30): patients with CHC and coinfection with HHV; group 2 (*n* = 67): only CHC without HHV. Values are the detection rates (**a**, **c**) or means ± SEM (**b**), * *p* < 0.05 between groups.

The presence of HCV RNA plus and minus strands in three substrates—sera, PBMC, and the liver—were determined (Figure 2a). Statistically significant differences were found between the patients of groups 1 and 2 in the frequency of HCV RNA detection in the blood sera: in group 1, the frequency of viral RNA (plus strands) exceeded the frequency in group 2 (*p* < 0.05). The average concentrations of core protein in the blood serum were also higher in group 1 (*p* < 0.05, Figure 2b). The groups did not differ in the frequency of genotype 1b HCV occurrence (Figure 2a), which is considered to be the most aggressive in comparison with other genotypes.

When comparing the spectrum of antibodies to HCV proteins in the blood sera of the patients, we found that, in group 2, antibodies to HCV NS5 were less frequently detected compared with in group 1 (*p* < 0.05, Figure 2c, Table 3). The average activity of aminotransferases did not differ in the groups of patients; however, the patients with permanently normal ALT levels were twice as many in group 2 as in group 1 (*p* < 0.05, Table 3). The percentages of patients with severe necroinflammatory lesions (histology activity score = 3) were similar in both groups. However, the proportion of patients with advanced stages of fibrosis and cirrhosis (F3 and F4, numerous septa without cirrhosis and cirrhosis) was two-times higher among group 1 patients compared to group 2, but the differences did not reach statistical significance.

**Figure 3:**
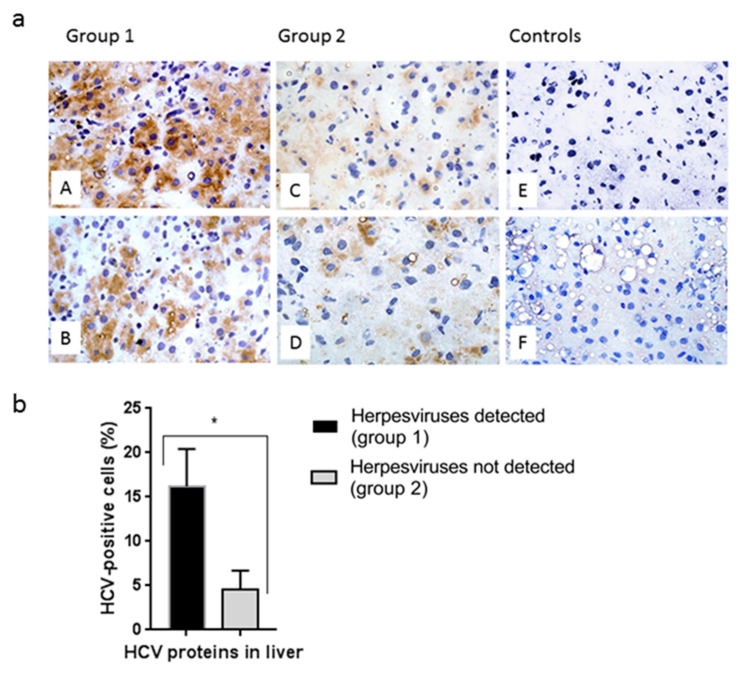
Comparative analysis of HCV proteins in hepatocytes of patients with chronic hepatitis C coinfected or not infected with herpesviruses. (**a**) Immunohistochemical detection of HCV proteins in cryostat liver sections using a mixture of mAbs to the core, NS3, NS4A/B, and NS5A proteins (A–D, F); E—mAb to HBsAg (negative control). A–E: patients with CHC, F: patient with steatohepatitis (negative control). Note more intensive cytoplasmic staining (brown) in group 1—HHV detected (A, B) compared with group 2—HHV not detected (C, D). No immunoreactivity was observed in the controls (E, F). The cell nuclei were stained with hematoxylin (blue). Magnification, × 400. (**b**) The relative number of HCV-containing liver cells in patients with CHC, depending on the detection of herpesviruses. Values are the means ± SEM (b), * *p* < 0.05 between groups.

Cryostat sections of liver biopsies from 12 patients in group 1 and 32 patients in group 2 were stained with mAbs to HCV proteins to detect infected cells. Immunohistochemical staining showed an increase in the proportion of HCV-containing hepatocytes in group 1 patients (Figure 3a,b). More intensive cell staining was also observed in group 1 patients (Figure 3a).

Thus, the data obtained showed that coinfection with herpesviruses in patients with CHC significantly aggravated the course of chronic hepatitis in terms of the histological, virological, and biochemical parameters.

## 4. Discussion

According to WHO data [23], viral hepatitis caused 1.34 million deaths in 2015, a number comparable to the deaths caused by tuberculosis and higher than those caused by HIV. Globally, in 2015, an estimated 257 million people were living with chronic HBV infection, and 71 million people were living with chronic HCV infection. In the Russian Federation, according to Russian Agency for Health and Consumer Rights data, 61.9 thousand cases of chronic viral hepatitises were registered in 2018, and, as for etiological structure, chronic hepatitis C dominated for the first time. In 2018, its share was 77.6%, and the share of chronic hepatitis B was 21.5% [24] These data indicate that, despite the introduction of DAA into practical healthcare in 2011, curing more than 90% of patients with hepatitis C, the annual mortality rate from viral hepatitis remains high [25].

There is much less information on hepatitis caused by other, non-hepatotropic viruses. Retrospective epidemiological analysis of morbidity based on the official statistics in 2009–2018 in the Russian Federation demonstrated that the share of acute and chronic hepatitis of unspecified etiology in the structure of morbidity of the total population accounted for 5.57% [26]. Researchers demonstrated that immunosuppressed patients and patients after liver transplantation often (up to 34% of cases) have hepatitis of herpesvirus etiology [6].

Liver lesions are described in the case of herpesvirus infections in adults and children, including infectious mononucleosis [27,28]. Many questions connected with the role of human herpesviruses in the occurrence, progression, and outcome of inflammatory liver diseases remain poorly understood. In this regard, it is of interest to determine whether HHV causes liver inflammation in immunocompetent individuals.

The analysis of three HHVs in this study demonstrated that the DNA of EBV, CMV, and HHV-6 was found in the PB in patients with both viral hepatitis B and C (in every seventh patient, 13.5%) and in the case of hepatitis of unspecified etiology (in every 10th patient, 10.1%).

EBV was detected in the PB more often than other HHV—in more than half of the cases (18/31, 58%, Table 1). EBV was also detected in the liver in 4/62 patients (6.5%) with hepatitis C (Table 2). EBV infection of the patients with viral hepatitis was also reported by researchers from Egypt, where the highest hepatitis C infection rate is registered [29]. EBV DNA was detected in 29% of patients with chronic hepatitis C, which was significantly higher than in the control group of individuals without viral hepatitis (7.7%) [30]. The authors stated that the levels of the ALT and AST were significantly higher in patients coinfected with EBV and HCV compared with in patients with EBV and HCV monoinfections [31]. Additionally, cases of acute hepatitis and fulminant hepatitis associated with EBV in immunocompetent patients were described [32,33,34].

B-lymphocytes are the most sensitive to EBV, although, recently, the prevailing opinion has been that the primary virus target is the oropharynx epithelial cells, in which the virus proliferates after oral transmission and is then transferred to B-cells. According to an alternative opinion, B-cells, infiltrating the oropharynx mucosa, infect epithelial cells. EBV is known to be found in some T- cell and NK (natural killer) tumors [35]. Thus, EBV infection is not restricted by the B-cell population and may also cause infection of other blood cells, and epithelial cells. Researchers may assume that EBV is able to participate in liver inflammation either by directly affecting the hepatocytes, or by acting indirectly through an immune-mediated route with participation of the immune cells and factors.

When studying the 68 liver biopsy samples obtained from the patients with a diagnosis of “liver disease of unknown etiology”, EBV DNA was detected in seven samples (10.3%) and immunohistochemically showed the existence of CD3+ and CD8+ lymphocytes infiltrating the liver [36]. Researchers assumed that HCV may cause EBV reactivation in latently infected lymphocytes, infiltrating the liver, and leading to liver damage. Liver disease may be a manifestation of chronic EBV infection with frequent reactivation and a constant moderate or low level of viral load [37].

In our study, EBV infection was detected not only in the case of hepatitis C but also in the case of coinfection with hepatitis B virus (HBV) in 9% of patients in the PB (Table 1). Previously, the description of severe acute hepatitis B associated with EBV was provided in the work S. C. Rao et al. [38]. The patient had jaundice, hyperbilirubinemia, increased transaminases, and coagulopathy.

Special attention should be paid to the patients with liver cirrhosis. In our study, cirrhosis was found in 16 of the 85 examined patients with hepatitis (Figure 2), and cirrhosis was three-times more frequent among the HHV-coinfected patients. One patient coinfected with hepatitis C virus and EBV and HHV-6 died. J. Hu et al. (2019) retrospectively analyzed the data on 97 patients with liver cirrhosis, of which 36 patients (37%) had EBV DNA in the whole blood [39]. The patients had EBV transcripts in lymphocytes infiltrating the liver, and a high level of EBV DNA in PB, suggesting virus reactivation. Patients with EBV infection had elevated Child–Pugh scores and high levels of liver enzymes. The authors concluded that the patients with cirrhosis infected with EBV had more pronounced liver damage, which is associated with a poor prognosis.

Along with EBV, the patients with viral hepatitis had HHV-6 in PB (in 2–8% of samples). In the liver, HHV-6 DNA was detected much more frequently—in samples from 20/62 patients (32.3%) (Table 2). Previously, HHV-6 was also detected in materials from patients (children) with acute hepatitis, both with an established cause and with an unspecified etiology [40]. Other authors concluded that herpesviruses may also lead to inflammatory liver diseases. This conclusion is confirmed by the data, in which liver samples from 22 patients with fulminant or active hepatitis of unspecified etiology were analyzed: HHV-6 DNA was detected in nine patients (40.9%) [41].

HHV-6 (HHV-6A/HHV-6B) is replicated primarily in activated CD4+ T lymphocytes but is also detected in CD8+ T lymphocytes, NK cells, monocytes/macrophages, and a wide range of other cell types, including liver cells [42]. Direct cell infection and cytokines and chemokines induced by HHV-6 infection may affect the spectrum and function of immune system cells in the course of infection. HHV-6 is able to induce immunomodulation via various mechanisms, such as lytic infection of CD4+ and/or cytotoxic effector T cells, the violation of antigen-representing functions, and inflammation induction [43]. A study demonstrated that HHV-6 may induce CD4(+) and CD8(+) HHV-6-specific regulatory T cells (Treg) [44]. These results allowed the authors to suggest that HHV-6 uses Treg induction to evade the antiviral immune response and maintain immunosuppression in the infected organism.

New results were obtained when analyzing the clinical and virological data from the patients with hepatitis C (Table 3, Figure 2). HCV genomic RNA was 1.6-times more common, and the concentration of HCV core protein was 2.5-times higher in the blood serum of the patients with HHV compared with in patients with hepatitis without HHV. More hepatocytes infected with HCV were found in the livers of patients with HHV after staining with mAbs to HCV core and nonstructural proteins. (Figure 3). Antibodies to the non-structural HCV NS5A protein were 1.5-times more often detected in the PB of patients with HCV/HHV coinfection (Figure 2).

Statistically significant differences in four virological indicators demonstrated more pronounced negative changes in the patients with coinfection. Thus, due to the high degree of correlation between the core protein and HCV RNA, the detection of increased concentrations of core protein in circulation indicates an active HCV infection [45]. HCV core protein demonstrated immunomodulatory activity and activated monocytes, macrophages, and Kupffer cells in the case of hepatitis C, which may cause the reactivation of latently infected herpesviruses; it was also demonstrated that the HCV core protein suppressed antiviral immunity [46].

The non-structural proteins of HCV play an important role in immune escape. The NS3/4A complex can inhibit RIG-I- and TLR4-mediated signaling to inhibit the activation of NF-κB [47]. NS5A modulates IRF-7-mediated interferon-α signaling [48]. NS5A is a multifunctional HCV protein that is involved in the HCV replication, interacts with various cellular factors, and affects the physiology of infected cells. We previously demonstrated that antibodies to this viral genome region are associated with a more severe progression of chronic hepatitis C [49].

Among the examined patients coinfected with viral hepatitis viruses and HHV, there were more men compared with patients without HHV (77% in comparison with 52%, *p* < 0.05), while there were less patients with consistently normal ALT levels (26% in comparison with 52%, *p* < 0.05). As female sex and normal values of ALT and AST in case of CHC are distinguished among the positive prognostic factors [50], our results indicate rather an unfavorable prognosis for the disease progression in patients coinfected with hepatitis viruses and herpesviruses.

The results of the analysis of the outcomes of the disease in 85 patients with hepatitis are of greater interest. After the treatment, condition improvements were observed in a larger number of patients in group 2 (without HHV); however, the differences did not reach statistical significance (Figure 1). At the same time, worsening was observed in a quarter of patients with hepatitis and HHV, while in the group without HHV—in 1 patient (1.3%)—and the differences between the groups were statistically significant.

Summarizing the data obtained, we concluded that HHV EBV, HHV-6, and CMV, detected in the PB and livers of patients with viral hepatitis and hepatitis of unspecified etiology, negatively affected the patient conditions and the disease outcomes. This was evidenced by (a) the increased frequency of HCV genomic RNA in the circulation; (b) a significant increase in the HCV core protein concentration in the PB; (c) an increased number of hepatocytes containing HCV proteins; (d) an increased level of antibodies to the HCV NS5A protein in PB; (e) the reduced number of patients with consistently normal ALT levels; (f) the worsening of conditions in a quarter of patients with herpesvirus infection in comparison with 1% in the group of patients without herpesvirus DNA in PB; (g) the development of liver cirrhosis being 2–3 times more common in patients with hepatitis B and C who were coinfected with HHV.

The more severe course of the disease in coinfection may be the result of various mechanisms that are not yet sufficiently understood. One of them may be intrahepatic replication of herpesviruses. Our data show the presence of HHVs DNA in archived liver samples of patients with hepatitis, but it is not established which cells contain the viral genome. Available evidence suggests that HHV may be present in both hepatocytes and nonparenchymal liver cells. So, in the liver of patient with fulminant hepatitis HHV-6 was localized in human hepatocyte nuclei, and an envelope antigen of this virus was detected in hepatocyte cytoplasm [41]. Additionally, in acute infection the main herpesviral producer (murine CMV model, mCMV) was the hepatocyte [51]. In latent herpesvirus infection, the main cellular site of mCMV latency and reactivation in the liver are liver sinusoidal endothelial cells (LSECs), which form a boundary surface for sensing of pathogens and interaction with lymphocytes [52]. In liver transplantation, HHV reactivation may be initiated in the sinusoidal endothelium, from which the virus can spread to hepatocytes, leading to cytopathogenic parenchymal infection and ultimately to graft failure [53]. One of the explanations for the increased frequency of HHV DNA detection in patients with CHC may be due to the reactivation of HHV triggered by HCV. Other data also support the conclusion that HHV may contribute to the development of hepatitis through immune-mediated effects. HCV RNA can be recognized by Toll-like receptor 3 or/and the helicase-mediated RIG-I pathway in the cytoplasm, resulting in transcriptional activation of molecular pathways and genes that reduce immunity, including trigger natural killer cells, increased cytotoxicity, and decreased regulation of proapoptotic factors and cytokines [54]. HHVs are recognized by other receptors (TLR2, 4, 8, and 9) and, infecting lymphocytes infiltrating the liver, additionally induce signaling pathways, which suppress the host immune system. Herpesvirus infection activate p38 mitogen-activated protein kinase (MAPK) signaling pathway and p38α activation facilitated HCV replication [55]. In the lymphocytes of patients with CHC, HHV can be both in an active and latent state, expressing a limited number of proteins. Latent proteins not only promote infection through activation of transcription factors like the AP-1, control expression of numerous cytokines, overexpression of BCL-2, but may also promote viral pathogenesis within the host [56]. At the same time, it is known that both HCV and HHV are able to suppress the cellular immune response, including interferon signaling, and to cause T cell exhaustion in chronic infections.

The limitation of the study is that HHV was detected in liver samples by one method–PCR, which allows determining the DNA of HHV, but does not show the presence of viral proteins. Currently, obtaining fresh liver samples is significantly difficult, since liver biopsies are rarely performed, and the histological activity of hepatitis and the stage of fibrosis are mainly determined by non-invasive methods in accordance with WHO recommendations.

## 5. Conclusions

The results obtained showed that in severe hepatitis and/or a poor response to specific drugs against viral hepatitis, it is advisable to evaluate the possible effect of human herpesviruses by PCR-determination of their DNA in whole blood or liver tissue (during liver biopsy or transplantation). Further studies engaging more patients are required to confirm the negative role of HHV in the viral hepatitis.

## Figures and Tables

**Table 1:** The frequency of herpesviruses detection in the peripheral blood of patients with different etiologies of liver damage.

Diagnosis	ICD-10 ^1^	Number of Patients, *n*	Number of Patients with Herpesviruses, *n* (%)				Frequency of Detection (%)
			CMV	HHV-6	EBV	HHV-6 +EBV	
Acute hepatitis A	B15	1	0	0	0	0	0
Acute hepatitis B and Chronic viral hepatitis B	B16.2 B16.9 B18.1	44	0	3 (6.8)	4 (9.0)	0	7 (15.9)
Acute delta-(super)infection in chronic hepatitis B and Chronic viral hepatitis B with delta-agent	B17.0 B18.0	10	0	0	0	0	0
Acute hepatitis C and Chronic viral hepatitis C	B17.1 B18.2	134	3 (2.2)	3 (2.2)	10 (7.5)	1 (0.7)	17 (12.7)
Chronic viral hepatitis B + Chronic viral hepatitis C	B18.1 + B18.2	1	0	0	0	0	0
Acute viral hepatitis, unspecified; Chronic viral hepatitis, unspecified; Unspecified viral hepatitis	B17.9 B18.9 B19	69	3(4.3)	0	4 (5.8)	0	7 (10.1)
Total		259	6 (2.3)	6 (2.3)	18 (6.9)	1 (0.4)	31 (12.0)

^1^ The International Statistical Classification of Diseases and Related Health Problems. 10th revision (ICD-10).

**Table 2:** Comparative analysis of the herpesvirus detection rates in various biological materials from patients with viral hepatitis C.

Clinical Materials		Frequency of Herpesvirus Detection, *n* (%)				
		CMV	HHV-6	EBV	HHV-6 + EBV	Total HHV
Peripheral blood	Whole blood (*n* = 79) ^1^	3 (3.8)	0	8 (10.1)	0	11 (13.9)
	Serum/plasma (*n* = 55) ^1^	0	3 (5.5)	2 (3.6)	1 (1.8)	6 (10.9)
	PBMC (*n* = 56) ^2^	1 (1.8)	1 (1.8)	7 (12.5)	0	9 (16.1)
Liver biopsy samples (*n* = 62) ^2^		0	20 (32.3)	3 (4.8)	1 (1.6)	24 (38.7) ^3^
Total samples (*n* = 242)		4 (1.7)	24 (9.9)	20 (8.3)	2 (0.8)	50 (20.7)

Notes: isolation of herpesvirus DNA for PCR was performed from freshly obtained ^1^ or archived thawed ^2^ materials; ^3^ the detection rate of herpesviruses in liver biopsies was statistically different from that in other materials (*p* < 0.005).

**Table 3:** Comparison of the demographic, histological, and serologic characteristics of patients with chronic hepatitis C depending on the detection of herpesvirus DNA in liver and blood cells.

Parameters		Group 1: HerpesvirusesDetected (*n* = 30) *n* (%) or Mean ± SEM	Group 2: Herpesviruses not Detected (*n* = 67) *n* (%) or Mean ± SEM	Statistical Significance
Age (years)		31.5 ± 2.2 ^1^	30.1 ± 1.5	*p* > 0.05
Gender—women proportion		7 (23.3) ^2^	32 (47.8)	*p* = 0.02
Illness duration (months)		60.6 ± 13.6	65.0 ± 15.4	*p* > 0.05
Intravenous drug users		13 (43.3)	21 (31.3)	*p* > 0.05
Serum aminotransferase level	AST (U/L)	39.5 ± 3.2	63.3 ± 7.0	*p* > 0.05
	ALT (U/L)	56.2 ± 13.8	45.3 ± 10.1	*p* > 0.05
	Continuously normal ALT (<40 U/L)	**8 (26.7)**	**35 (52.2)**	***p* = 0.03**
Grading and staging of the liver lesions (METAVIR)	Histology activity A3 (severe) Fibrosis scoring F ≥ 3 (numerous septa and cirrhosis)	5 (16.7) 7 (23.3)	12 (17.9) 7 (10.4)	*p* > 0.05 *p* > 0.05

Notes: ^1^ mean values ± standard errors; ^2^ frequency of occurrence of the parameter; categorical variables were tested using χ2 and Fisher’s exact tests; continuous variables analyzed using a two-tailed Student’s *t*-test; statistically significant differences between groups (*p* < 0.05) are highlighted in bold.

## Data Availability

The study did not report any data.
